# The effect of tiotropium therapy on markers of elastin degradation in COPD

**DOI:** 10.1186/1465-9921-10-12

**Published:** 2009-02-25

**Authors:** Shuren Ma, Yong Y Lin, Lori Tartell, Gerard M Turino

**Affiliations:** 1 James P. Mara Center for Lung Disease, St. Luke's-Roosevelt Hospital Center, 1000 Tenth Avenue, New York, NY 10019, USA

## Abstract

**Background:**

Desmosine and Isodesmosine (D/I) are cross-linking amino acids which are present only in mature elastin. Changes in their concentration in body fluids indicate changes in elastin degradation and can be a reflection of tissue elastase activity. This study was undertaken to determine whether continuous therapy with the long-acting bronchodilator Tiotropium bromide (TTP) could result in reductions in D/I as measured by mass spectrometry in plasma, urine and sputum.

**Methods:**

Twelve not currently smoking patients with chronic obstructive pulmonary disease (COPD), never on TTP, were selected for study. Levels of D/I, along with measurements of FVC, FEV_1 _and FEV_1_/FVC. were determined before starting TTP daily, and then one and two months after.

**Results:**

D/I decreased in plasma (10 of 12 patients), in sputum all (12 of 12), and in the percentage of free D/I in urine (10 of 12). Most patients showed slight increases in FVC and FEV_1 _percent predicted over two months.

**Conclusion:**

The results are consistent with an effect of prolonged bronchodilitation by anti-cholinergic blockade to also result in reduced lung elastin degradation.

## Background

In chronic obstructive pulmonary disease (COPD) tissue elastin injury[1] and depletion[2] have been demonstrated in lung parenchyma. Recently, techniques for detecting and quantifying elastin degradation in body fluids have advanced in specificity, sensitivity and accuracy by the use of mass spectrometry[3]. Desmosine and Isodesmosine (D/I) are cross-linking amino acids which are present only in mature elastin so that changes in their concentration in body fluids are a reflection of elastin degradation and would therefore not be a measure of elastin synthesis from precursors[4]. The use of these analytical techniques has resulted in the demonstration that patients with COPD related to smoking or the inherited deficiency of alpha-1 antitrypsin (AATD) have elevated levels of D/I in blood plasma, sputum and as a free unconjugated component in urine[5].

The use of these markers of lung elastin degradation in disease offers the prospect of evaluating levels of D/I as indicators of possible efficacy of therapeutic interventions.

The long acting bronchodilator TTP has been shown to reduce hospitalizations and the frequency of exacerbations in large patient populations of COPD [6-8]. TTP has also been shown to reduce the level of lung hyperinflation in COPD[9]. Previous studies have suggested that blocking acetylcholine may have effects on inflammatory mediators and smooth muscle growth factors. Such effects may be reflected in lung matrix injury with respect to elastin degradation [10,11]. This study examines that possibility in 12 patients with COPD studied over a 2-month interval prior to the initiation of TTP therapy and continuing daily TTP for a period of 2 months. The results indicate significant reductions in D/I in the majority of patients so treated.

## Materials and methods

Preparation of specimens of urine, plasma and sputum by liquid chromatography (LC), mass spectrometry (MS) has been described previously[2,5]. Analysis of urine utilized aliquots from 24-hour urine collections in each patient. Each sample of plasma, urine and sputum was analyzed in triplicate and their mean values and standard deviations calculated. All standard deviations are below ± 10%.

Twelve patients with clinically stable COPD were selected for study. All patients had physiologic evidence of airway obstruction. Eleven had a history of smoking for at least 10 years but were not smoking at the time of the study and had stopped smoking over 5 years before the study. One patient with alpha-1 antitrypsin deficiency had never smoked. Two patients had homozygous-Z phenotype alpha-one antitrypsin deficiency (ATTD). Patients were categorized as GOLD stages 2 and 3[12]. None of the patients had been administered TTP prior to the beginning of the study. Patients remained on their existing medical regimens. None were on oxygen or were undergoing a rehabilitation program. If they were taking any anti-cholinergic bronchodilators prior to the study, that medication was stopped when TTP therapy began. 18 μg of TTP was administered every 24 hours. No patient had the addition or deletion of steroid inhalants during the 2-month period of study.

Spirometric indices were FEV_1_, FEV_1_/FVC and FVC, measured prior to and after 1 and 2 months of therapy. D and I in urine, plasma and sputum were measured by LC/MS prior to the study and at 1 month and 2 months after the beginning of the study. Statistical analysis was carried out by two-tailed T test (Graph Pad Prism 4 software) p < .05 statistical significance.

## Results

**Figure 1:**
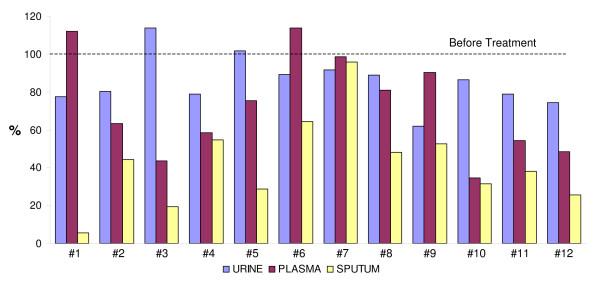
**Shown are the percentages reduction in the summed levels of Desmosine and Isodesmosine in 24-hour urine, plasma and sputum for each of 12 patients after 2-months of Tiotropium administration**. The dotted line represents the pre-administration level for each patient.

Decreases in D/I levels were observed in the free componend of urine (10 of 12 patients), in plasma (10 of 12) and in sputum (all 12 patients) which is consistent with reductions in mature elastin degradation following the initiation of tiotropium therapy (see Table 2 and Figure 1). The percent reductions in D/I shown in Fig. 1 were calculated for each patient as the ratio derived from the difference between the pre-treatment levels of D/I and the levels at month 2 divided by the pre-treatment level and expressed as percent reduction at 2 months. The calculated percent decreases in D/I levels after TTP treatment showed the decreases beginning after one month, with further decreases in the second month. These reductions at 2 months averaged 15% (range 9–38%) in urine; 27% (range 2–65%) in plasma and 58% (range 4–98%) in sputum. Mean reductions in each body fluid for the 12 patients were statistically significant at p < .005.

Tables 1, 2 and 3 show the mean values and standard deviations for all 12 patients for levels of D/I at base line and after 2 months of tiotropium therapy and the statistical significance for the changes in urine, plasma and sputum. A paired T-test was used to test the null hypothesis that the mean value at baseline for all 12 patients was equal to the mean value at 2 months for all 12 patients. Separate analysis was performed for urine, plasma and sputum. The accepted level of significance was equal to .05.

**Table 1:** Age, Gender, Race and Pulmonary Function of study patients

**PATIENT NUMBER**	**AGE**	**GENDER**	**RACE**	**STUDY**	FVC** %Pred	FEV_1_** %Pred	FEV_1_/FVC** %
-1-	61	M	C*	Pre-Tio 1 mo post 2 mo post	77 91 77	47 53 47	46 44 46

-2-	79	F	C*	Pre-Tio 1 mo post 2 mo post	97 96 106	64 72 73	49 56 51

-3-	65	F	AA*	Pre-Tio 1 mo post 2 mo post	106 96 108	74 65 66	54 53 48

-4-	63	F	C*	Pre-Tio 1 mo post 2 mo post	92 107 100	41 52 45	46 48 46

-5-	65	F	H*	Pre-Tio 1 mo post 2 mo post	70 84 84	67 84 82	75 79 78

-6-	65	M	A*	Pre-Tio 1 mo post 2 mo post	56 49 51	49 49 52	66 75 78

-7-	64	M	C*	Pre-Tio 1 mo post 2 mo post	59 69 64	41 47 41	51 51 48

-8-	39	M	C*	Pre-Tio 1 mo post 2 mo post	52 69 62	24 30 29	30 35 38

-9-	57	F	H*	Pre-Tio 1 mo post 2 mo post	93 87 83	55 55 53	47 51 51

-10-	69	M	AA*	Pre-Tio 1 mo post 2 mo post	58 82 109	30 39 54	39 36 38

-11-	66	F	C*	Pre-Tio 1 mo post 2 mo post	97 103 90	62 60 58	49 45 48

-12-	59	M	C*	Pre-Tio 1 mo post 2 mo post	110 91 102	72 69 71	50 57 53

* AA = African-American

C = Caucasian

H = Hispanic

A = Asian

** FVC%Pred = Forced Vital Capacity % of predicted

FEV-1%Pred = Forced Expiratory Volume in 1 second % of predicted.

FEV-1/FVC% = absolute ratio as percent.

**Table 2:** Effect of Tiotropium treatment on levels of desmosine and Isodesmosine

**PATIENTS**		**URINE** **(ug/g creatinine)**		**PLASMA** **(ng/ml)**	**SPUTUM** **(ng/ml)**
				
		**FREE**	**F/T(%)**		
-1-	0 month 1 month 2 month	6.57 4.99 5.29	49.6 48.5 38.5	0.42 0.37 0.47	0.92 0.15 0.05

-2-	0 month 1 month 2 month	9.50 5.91 7.50	40.8 41.9 32.7	0.71 0.67 0.45	0.77 0.19 0.33

-3-	0 month 1 month 2 month	3.90 5.37 4.45	35.1 38.1 39.9	0.71 0.54 0.33	0.52 0.26 0.10

-4-	0 month 1 month 2 month	5.14 4.82 6.91	45.0 42.3 32.5	0.77 0.40 0.45	0.33 0.23 0.18

-5-	0 month 1 month 2 month	7.54 4.95 4.68	46.2 51.4 47.1	0.73 0.57 0.55	0.49 0.16 0.14

-6-	0 month 1 month 2 month	3.31 3.72 3.05	36.2 34.2 31.4	0.44 0.40 0.50	0.05 0.01 0.03

-7-	0 month 1 month 2 month	3.40 5.38 4.79	40.9 40.2 37.4	0.62 0.63 0.61	0.23 0.30 0.22

-8-	0 month 1 month 2 month	3.76 3.48 3.15	39.4 35.3 35.0	0.52 0.39 0.42	0.27 0.29 0.13

-9-	0 month 1 month 2 month	6.61 5.26 4.71	63.2 43.0 39.1	0.51 0.64 0.46	0.19 0.22 0.10

-10-	0 month 1 month 2 month	7.59 5.98 4.87	50.7 46.6 43.9	0.75 0.46 0.26	0.19 0.12 0.06

-11-	0 month 1 month 2 month	5.67 4.71 4.97	51.0 45.3 40.2	0.61 0.38 0.33	0.22 0.08 0.08

-12-	0 month 1 month 2 month	5.83 5.13 5.55	53.4 48.9 39.7	0.67 0.34 0.33	0.52 1.07 0.13

Normal Subjects[5] (n-13)		2.52 (± 0.53)	19.0 (± 2.0)	0.19 (= + 0,01)	none

**Table 3:** Mean changes in desmosine and Isodesmoeine after two months of Tiotropium therapy

**BODY FLUID**	**N**	**LEVELS (mean ± SD)**		**P VALUE**
			
		**AT BASELINE**	**AT 2 MONTHS**	
Plasma ng/ml	12	0.62 ± 0.12	0.43 ± 0.10	0.0037

Urine (free/total %)	12	45.96 ± 8.11	38.37 ± 4.43	0.0044

Sputum ng/ml	12	0.39 ± 0.26	0.13 ± 0.02	0.0035

After 2 months of treatment, larger decreases in D/I levels were observed in sputum and plasma than in urine. The response is not always uniform in urine, plasma and sputum. Two patients (#3 and #5) failed to show decreases in urine but showed decreases in their plasma and sputum and another two patients (#1 and #6) not decreasing in plasma showed decreases in urine and sputum (see figure 1).

Overall results indicate that all 12 COPD patients responded to prolonged TTP treatment with some decrease in lung elastin degradation as measured in one of the body fluids analyzed.

Spirometrically, most patients showed slight increases in FVC and FEV_1 _percent predicted with usually little change in the FEV_1_/FVC ratio (see Table 1).

## Discussion

D/I measured by mass spectrometry has the advantage of identifying and quantifying these cross-linking amino acids of elastin which are present only in mature elastin and not present in the elastin precursor tropoelastin. As such, the levels of D/I in plasma, urine and sputum are reflecting mature elastin breakdown. Since mature elastin cleavage requires the activity of specific elastases, reductions in levels of D/I in body fluids most probably are reflecting decreases in the specific activity or the concentrations of elastases in the tissue milieu. The prominent tissue elastases which have been identified in bronchi and parenchyma of the lung are neutrophil elastase[13] and metalloproteases[14], of which metalloproteases 1[15], 2[16], 8,9[17-19] have been identified in COPD. The decreases of D/I in urine, plasma and sputum in the majority of patients in this study after initiating long-acting anticholinergic therapy is consistent with reduction in elastase activity.

It has been our premise that the content of free (unconjugated) D/I occurs as a result of elastase activity in neutrophils and macrophages in blood and tissues, which can degrade elastin fragments prior to excretion in urine and therefore may be an indication of stimulation of neutrophils and macrophages by a heightened inflammatory state of patients with COPD as indicated by increased inflammatory markers detected in COPD [20,21]. The reduction in the free total excretion ratio of D/I with Tiotropium therapy would be consistent with an anti-inflammatory effect of the therapy. This anti-inflammatory effect could occur from several mechanisms.

Improved clearance of bronchial secretions could occur consistently with decreased airway obstruction, which could reduce bacterial colonization with reductions in virulence and bacterial species.

Reducing airway obstruction and the state of lung hyperinflation may have a beneficial effect through a reduction in tissue stretch. Prior work has suggested that mechanical forces in the airways and surrounding alveolar structures may impose cellular and cytokine responses that are pro-inflammatory and stimulate bronchial smooth muscle reactivity [22,23]. In support of this concept, pro-inflammatory cytokines are increased in ventilator-induced lung injury and may be elevated in distended lung tissue[24]. Also, it has been shown that cycling mechanical stretch can profoundly affect gene expression [22,23].

TTP, which blocks acetylcholine receptors has been demonstrated to inhibit allergin-induced airway remodeling in a Guinea pig model of ongoing asthma[10]. Thus, endogenous acetylcholine may be an important mediator in airway smooth muscle remodeling in asthma, a process which also has involved chronic inflammatory stimuli[11]. It is worthy of consideration that such mechanisms involving the role of acetylcholine could be involved in COPD as well as asthma and that blocking acetylcholine activity might have anti-inflammatory effects.

Additional studies have been reported which show that tiotropium can inhibit allergen-induced airway remodeling in a Guinea pig model of allergic asthma[25]. Also, tiotropium has been shown to suppress acetylcholine-induced release of chemotactic mediators in vitro in neutrophils and macrophages and specifically LTB4[26]. However, measurements of sputum and serum markers of inflammation such as CRP and IL-6 have not been reduced in patients with COPD treated for 12 months with tiotropium[27]. Further study of TTP and other inflammatory markers in COPD seem warrented.

It should be noted that measurements of (D/I) in plasma and urine may be reflecting elastin degradation derived from elastin sources other than the lung per se, such as blood vessels or skin. D/I in sputum, however, should be reflecting only elastin degradation from lung tissue and therefore may be the most sensitive index of a therapeutic effect[28]. Also, the presence of D/I in sputum is an indictor that lung elastin is in flux, although the contribution to plasma or urinary levels from that source cannot be determined. In this regard, induced sputum from normal subjects has no detectable D/I[3].

This anti-inflammatory response to TTP, as demonstrated by measurements of D/I are consistent with the preliminary result of a reduction in FEV_1 _loss at the end of one year of follow-up in patients receiving TTP therapy[29]. Also, the results of this study are consistent with the previously reported reductions in COPD exacerbations and required hospitalizations in large cohorts of COPD patients [6-8].

The pre-TTP treatment levels of D/I were established with single measurements in plasma, urine and sputum in each patient. This may be of concern since fluctuations of single measurements in an individual might effect the final results. In this regard, prior data has been published from our laboratory[5] concerning the variation of repeat measurements in plasma in single individuals in a stable clinical state over days, weeks and months. The variability was maximally 15%. The results of the present study demonstrated consistent reductions in levels of D and I in plasma, urine and sputum which result is unlikely to be reflecting fluctuations in the measurements during a stable clinical state. Also, a recent study[30] in patients with AATD demonstrated increases in urinary desmosine at six months and 1 year and no decreases.

There were no clinical or spirometric characteristics which distinguished those patients who had the most marked reduction in D/I from those less responsive. Lack of correlation of D/I excretory patterns with clinical phenotype in COPD has been previously reported[31].

Patients #8 and #12" had AATD. Both have quite marked reductions in the sputum levels of D/I with modest reductions in urine and plasma.

AATD patients have been shown to have higher levels of D/I among COPD patients in general[5]. Whether the reduction in D/I in sputum or plasma and urine could be greater in AATD patients in general must await further study. The results of this study indicate the potential application of D/I as markers to evaluate therapeutic effects in COPD.

## Conclusion

Two months of therapy by anticholinergic blockage for bronchodilitation resulted in reduction in elastin degradation in most patients with COPD, suggesting an anti-inflammatory effect.

## Abbreviations

COPD: Chronic Obstructive Pulmonary Disease; D/I: desmosine and isodesmosine; LC: liquid chromatography; MS: Mass Spectrometry; FVC: Forced vital capacity; FEV_1_: Forced expiratory volume in one second

## Competing interests

The authors declare that they have no competing interests.

## Authors' contributions

SM performed liquid chromatography, mass spectronometric measurements of D/I and statistical analysis of data; YYL assisted with preparative procedures for chemical analysis of D/I and study planning; LT supervised patients selected for study and their participation in the study, including spirometry; GMT was involved in study planning, patient selection and a creating a draft manuscript. All authors participated in manuscript design and revisions and approved the final manuscript.
